# Single-neuronal cell culture and monitoring platform using a fully transparent microfluidic DEP device

**DOI:** 10.1038/s41598-018-31576-2

**Published:** 2018-09-04

**Authors:** Hyungsoo Kim, In-Kyu Lee, Kendra Taylor, Karl Richters, Dong-Hyun Baek, Jae Ha Ryu, Sang June Cho, Yei Hwan Jung, Dong-Wook Park, Joseph Novello, Jihye Bong, Aaron J. Suminski, Aaron M. Dingle, Robert H. Blick, Justin C. Williams, Erik W. Dent, Zhenqiang Ma

**Affiliations:** 1 Department of Electrical and Computer Engineering, University of Wisconsin–Madison, Madison, WI 53706 USA; 2 Neuroscience Training Program, University of Wisconsin-Madison, Madison, WI 53705 USA; 3 Department of Neuroscience, University of Wisconsin-Madison, Madison, WI 53706 USA; 4 Department of Biomedical Engineering, University of Wisconsin–Madison, Madison, WI 53706 USA; 5 School of Electrical and Computer Engineering, University of Seoul, Seoul, 02504 South Korea; 6 Department of Surgery, University of Wisconsin-Madison, Madison, WI 53706 USA

## Abstract

Dielectrophoresis using multi-electrode arrays allows a non-invasive interface with biological cells for long-term monitoring of electrophysiological parameters as well as a label-free and non-destructive technique for neuronal cell manipulation. However, experiments for neuronal cell manipulation utilizing dielectrophoresis have been constrained because dielectrophoresis devices generally function outside of the controlled environment (*i.e*. incubator) during the cell manipulation process, which is problematic because neurons are highly susceptible to the properties of the physiochemical environment. Furthermore, the conventional multi-electrode arrays designed to generate dielectrophoretic force are often fabricated with non-transparent materials that confound live-cell imaging. Here we present an advanced single-neuronal cell culture and monitoring platform using a fully transparent microfluidic dielectrophoresis device for the unabated monitoring of neuronal cell development and function. The device is mounted inside a sealed incubation chamber to ensure improved homeostatic conditions and reduced contamination risk. Consequently, we successfully trap and culture single neurons on a desired location and monitor their growth process over a week. The proposed single-neuronal cell culture and monitoring platform not only has significant potential to realize an *in vitro* ordered neuronal network, but also offers a useful tool for a wide range of neurological research and electrophysiological studies of neuronal networks.

## Introduction

Single-cell analysis has attracted an increasing amount of attention over the past decades, and paves the way for elucidating fundamental biological phenomena such as cellular processes and heterogeneities^1,2^. Of particular importance to the field of neuroscience, meticulous studies of single neurons and between spatially isolated neurons provide a better understanding of the dynamics of functional neuronal networks as well as their fundamental molecular and cellular mechanisms^3^. This research is essential to push forward personalized treatments of neurological disorders including epilepsy, Parkinson’s disease, Alzheimer’s disease, and other cognitive and motor disorders^4^.

To date, various cell manipulation techniques such magnetophoresis, optical tweezers, acoustic means, and dielectrophoresis (DEP), have been explored for the field of single-cell analysis^2,5–9^. Among these techniques, DEP, an electrokinetic phenomenon acting on polarizable particles in a non-uniform electric field, benefits from the fact that cells can be trapped, aligned and patterned without requiring additional elements (*i.e*. optical device, magnet and light source)^5,10–16^. Moreover, DEP provides a healthy environment for neurons to reside by incorporating electrode structures that are designed to minimize the electric field intensity^9^.

Nevertheless, the availability of DEP for realizing an *in vitro* cultured neuronal network is limited by the difficulty of the neuron cultures, which are highly susceptible to the properties of the physiochemical environment (*i.e*. pH, osmotic pressure, humidity, and temperature) and infection^17–20^. In addition, imaging the morphology and activity of cultured neurons using inverted microscope is often confounded by the use of non-transparent electrodes and substrate^17–19,21^.

Here, we propose an advanced single-neuronal cell culture and monitoring platform using a fully transparent microfluidic DEP device. This device consists of multi-electrode arrays (MEAs) made of indium-tin-oxide (ITO) and a polydimethylsiloxane(PDMS) microfluidic chip. To reduce the risk of culture contamination, the device was mounted inside an incubated microscope system. A target neuron was trapped and released sequentially by an array of ring-shaped electrodes arranged in a row to demonstrate the capabilities of the proposed system. Consequently, we were able to successfully culture and monitor single-neuronal cells over time. This advanced platform for trapping of single-neuronal cells and monitoring of its electrophysiological parameters enables novel and detailed neurological studies.

## Theory

### Dielectrophoresis

DEP is a translational motion of polarizable particles suspended in a medium induced by a non-uniform electric field^12,22^. The time-averaged DEP force on a particle in a non-uniform electric field can be expressed as

1 $${\overrightarrow{F}}_{DEP}=2\pi {R}^{3}{\varepsilon }_{m}{\rm{Re}}[{f}_{CM}(\omega )]\nabla {\overrightarrow{E}}_{rms}^{2}$$

where R is the radius of the particle, *ε*_*m*_ is the relative permittivity of the surrounding medium, Re[*f*_*CM*_(*ω*)] is the real part of the Clausius-Mossotti (CM) factor, ▽ is the del vector operator, and *E*_*rms*_ is the root-mean-square value of the applied electric field^12^. For the case of a spherical, homogeneous particle of permittivity *ε*_*p*_, the CM factor which describes the effective polarizability of the particle which varies with the applied frequency^23^ is given by

2 $${f}_{CM}(\omega )=\frac{{\varepsilon }_{p}^{\ast }-{\varepsilon }_{m}^{\ast }}{{\varepsilon }_{p}^{\ast }+2{\varepsilon }_{m}^{\ast }}$$

where ${\varepsilon }_{p}^{\ast }$ and ${\varepsilon }_{m}^{\ast }$ are the complex permittivities of the particle and the medium, respectively, with ${\varepsilon }_{p}^{\ast }={\varepsilon }_{p}-j\frac{{\sigma }_{p}}{\omega }$, and ${\varepsilon }_{m}^{\ast }={\varepsilon }_{m}-j\frac{{\sigma }_{m}}{\omega }$ where *ε*_*p*_ and *ε*_*m*_ are the permittivities of the particle and the medium, respectively, *σ*_*p*_ and *σ*_*m*_ are the conductivities of the particle and the medium, respectively, and ω is the angular frequency of the applied electric field. The real part of the CM factor (Re[*f*_*CM*_(*ω*)]) has a value between −0.5 and 1 and determines the direction of the DEP force. When the particles that are more polarizable than the surrounding media, the Re[*f*_*CM*_(*ω*)] is positive and the particles are attracted to the regions of electric field intensity maxima, positive dielectrophoresis (pDEP), whereas when the particles that are less polarizable than the media, Re[*f*_*CM*_(*ω*)] is negative and the particles move toward electric field intensity minima (*i.e*. repelled from field maxima), negative dielectrophoresis (nDEP). For neuroscience applications, the nDEP is better suited as it makes possible the use of commonly used neuronal cell culture media due to the conductivity and permittivity of the media being higher than those of neurons^10,11,19^ as well as allows neurons to reside in a healthy environment by attracting the neurons to the region of electric field intensity minima.

## Results and Discussions

### Device modeling and simulation

To verify the feasibility of the proposed DEP device for single neuronal cell manipulation, the strength of the electrical field and the direction of the DEP force over the device including electrodes were numerically solved using finite element simulation software (Comsol Multiphysics 4.2, Comsol Ltd). The amplitude and frequency of the applied voltage in this simulation were 8 V_pp_ and 10 MHz, respectively. For the designed ring-shaped electrodes, we presumed the electric field distribution to be cylindrically symmetric in any plane orthogonal to the plane of the array of electrodes.

For the simulation, various physical parameters of the structure and a dipolar model of the DEP force were established. The parameters of the neuronal cell and medium were as follows: the radius of the cell: 5 µm, permittivity of the cell: 80, cytoplasm permittivity: 7.1 × 10^−10^ F/m, cytoplasm conductivity: 0.75 S/m, membrane permittivity: 1.8 × 10^−12^ F/m, membrane conductivity: 1 × 10^−7^ S/m and medium permittivity: 7.1 × 10^−10^ F/m^14,19,24–26^. As for the boundary conditions, applied AC electric potentials were on the ring-shaped electrodes and the outer surfaces were set to electrical insulation.

**Figure 1:**
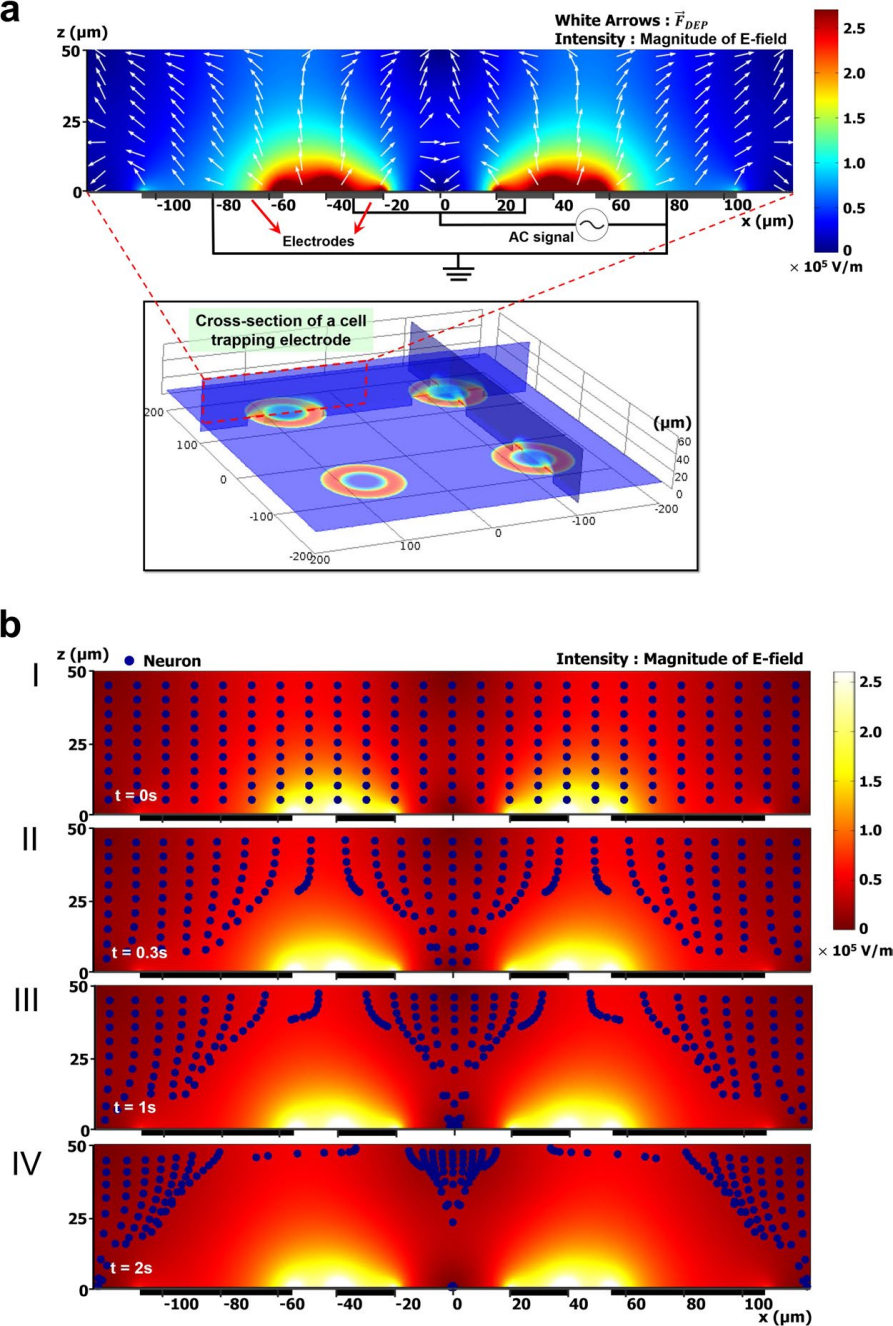
Schematic illustrations of trap electrode arrays and its cross-sectional view with numerical simulation results. The color bar shows the electric field intensity (in V/m) for an applied AC signal. (**a**) Distribution of the electric field magnitude (in V/m), based on an applied potential of 8 Vpp at 10 MHz, is shown for each trap electrode inside the fluidic channel with color-scale plot. The white arrows, normalized vectors, indicate the direction of the dielectrophoretic force. The intensity of the applied electric field is maximal in close proximity to the edge of each ring-shaped electrode and is reduced to its minimum value at the center of the trap zone. (**b**) Motion trajectories of neurons with a radius of 5 µm under the distribution of applied electric field magnitude (in V/m). Numerals I-IV correspond to time: (I) Initial distribution of neurons in the domain, (II) position of the neurons after 0.3 s, (III) 1 s, and (IV) 2 s.

Figure 1a shows the distribution of the electric field magnitude (E^2^) for each trap electrode with an applied signal of 8 Vpp at 10 MHz. As can be seen in this figure, the magnitude of the electric field changed over the position (x-axis), with a minimum in the center of the ring-shaped trap electrode and maximum at the edge of electrode in the gap between the trap electrode and surrounding counter electrode. Dielectrophoretic forces (white arrows) are directed toward the center of the ring-shaped electrode and repellent forces are displayed near the surrounding electrodes. This simulation result indicates that invisible trap formed at the center of the electrode with low electric field magnitude.

The numerical simulation results of the neuron motion tracking at each instant are illustrated in Fig. 1b. Neurons were modeled as blue particles with a radius of 5 µm and distributed uniformly at the initial stage (I) of the simulation. The blue particles placed near the ring trap were driven toward regions of low field strength and collected in the center of the ring trap. However, the particles placed outside of the ring trap moved upwards, repelled by the repulsive force over time. This simulation indicates that our microfluidic DEP device is suitable for single cell manipulation.

### Single-neuronal cell manipulation

**Figure 2:**
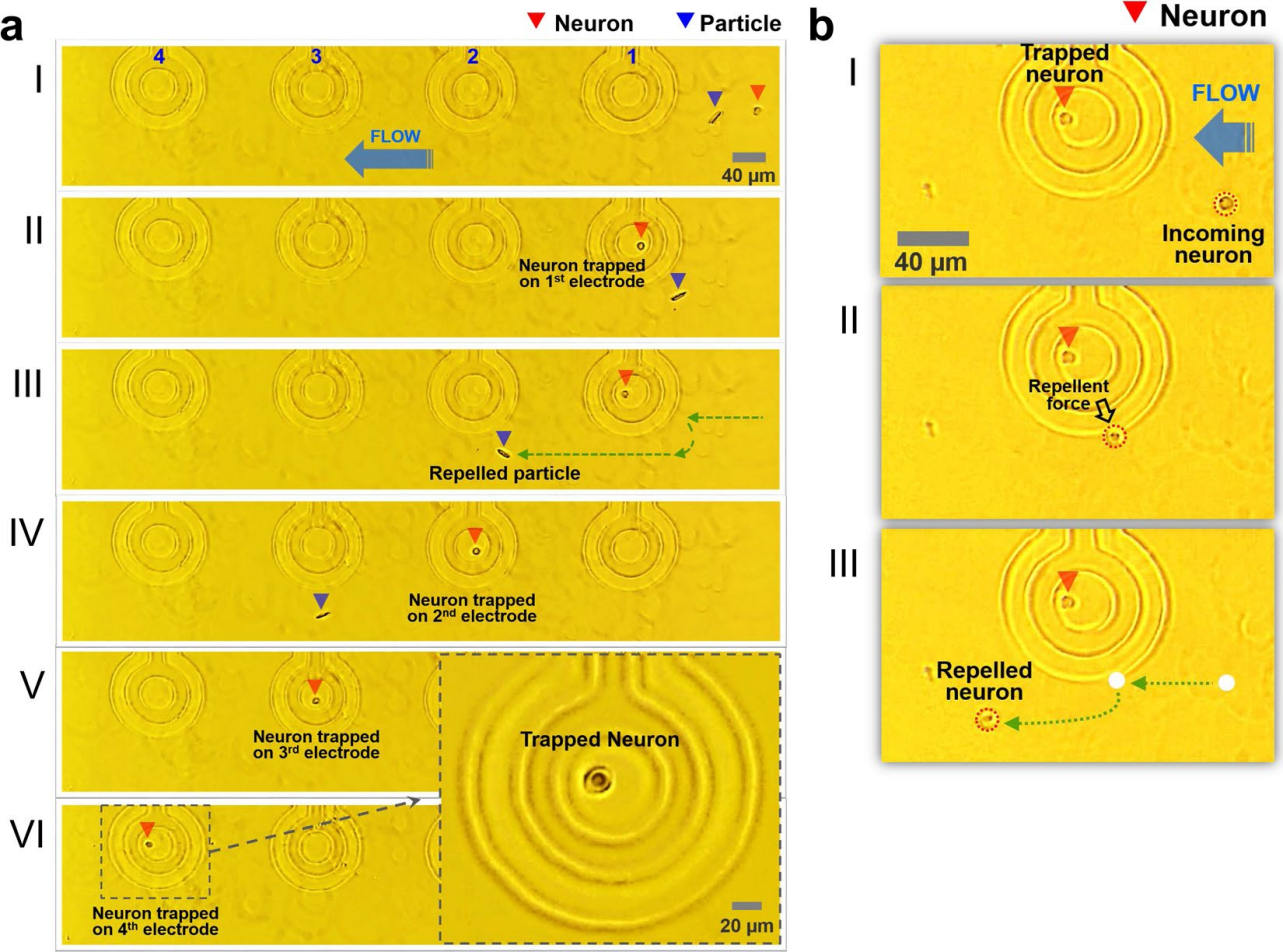
Recorded images of single-neuronal cell manipulation on the array of ring-shaped traps. (**a**) Incoming neuron (I) entering the 1^st^ trap. (II) The neuron is then immobilized in the 1^st^ trap electrode against a fluid flow. (III) While the neuron is trapped, a repelled particle continues to move in the flow of media. (IV) The released neuron is captured again in the 2nd trap. (V and VI) The neuron is trapped in the 3^rd^ and the 4^th^ ring trap in turn. (**b**) Bouncing motion of the neuron subject to a repulsive force. While the target neuron was trapped in the desired electrode, an incoming neuron was repelled by DEP force. When the incoming neuron reached the outside of the electrode, the repulsive force pushed the neuron out of the ring. Video is included in Supplementary Materials (Movie S1).

To demonstrate the performance of our proposed fully-transparent microfluidic DEP device, single-neuronal cell manipulation was conducted as shown in Fig. 2. The cell trapping process was carried out inside an incubator and monitored with a built-in CCD camera (DS-Qi1, Nikon, Inc.). The single-neuronal cell trapping process is illustrated in Fig. 2a. The neuronal cells were trapped and released sequentially by an array of ring electrodes arranged in a row. Details of the neuronal cell positioning process are as follows: (I) A target neuron (red arrowhead) flows to the 1^st^ electrode. (II) When the target neuron comes in proximity to the 1^st^ electrode, the 1^st^ electrode is energized and the neuron is immobilized in the center of the 1^st^ trap site. (III) Non-cellular particles (blue arrowhead) are repelled by the 1^st^ electrode and keep flowing while the trapped neuron remains at the 1^st^ trap site (green arrows). (IV) The trapped neuron is released by turning the 1^st^ electrode off, travels with the flow of the media, and then is trapped again in the center of the 2^nd^ trap site. (V-VI) The neuron is then subsequently released and trapped in turn by the 3^rd^ and 4^th^ trap sites.

We also observed neurons being repelled from the electrode. The images in Fig. 2b show the bouncing motion of the neuron which is subject to a repulsive force induced by the nDEP. (I and II) While the target neuron was immobilized in the ring trap (red arrowhead), another neuron comes in close proximity to the ring trap and it appears to have bounced off the invisible wall created by the repulsive force (black arrow). (III) The repelled neuron travelled along the invisible wall carried by the flowing media, while the target neuron stays in the trap. The trapping and bouncing motion of the neuron was in concordance with the particle trajectory simulation results (Fig. 1b). Video of single-neuronal cell manipulation recorded through the transparent electrode sites can be seen in the Supplementary Materials (Movie S1).

### Single-neuronal cell culture, monitoring and imaging

After the cell trapping process, the media was aspirated and replaced with fresh culture media to remove any redundant neurons and cellular debris remaining in the microfluidic chamber and deliver nutrients to the trapped neurons. The fluid flow was shut down and the system was stabilized for 5 min. Then, the nDEP forces were turned off so that the trapped neurons levitating above the electrode plane were released from its levitated position and plated down for culture. During the cell culture period, we changed the culture media once a day to supply nutrients to the neurons by aspirating away approximately half of the media and replacing the amount removed with fresh media. The cultivation images were recorded by a CCD camera integrated inside the incubator. Red LED illumination was used for phase contrast imaging.

**Figure 3:**
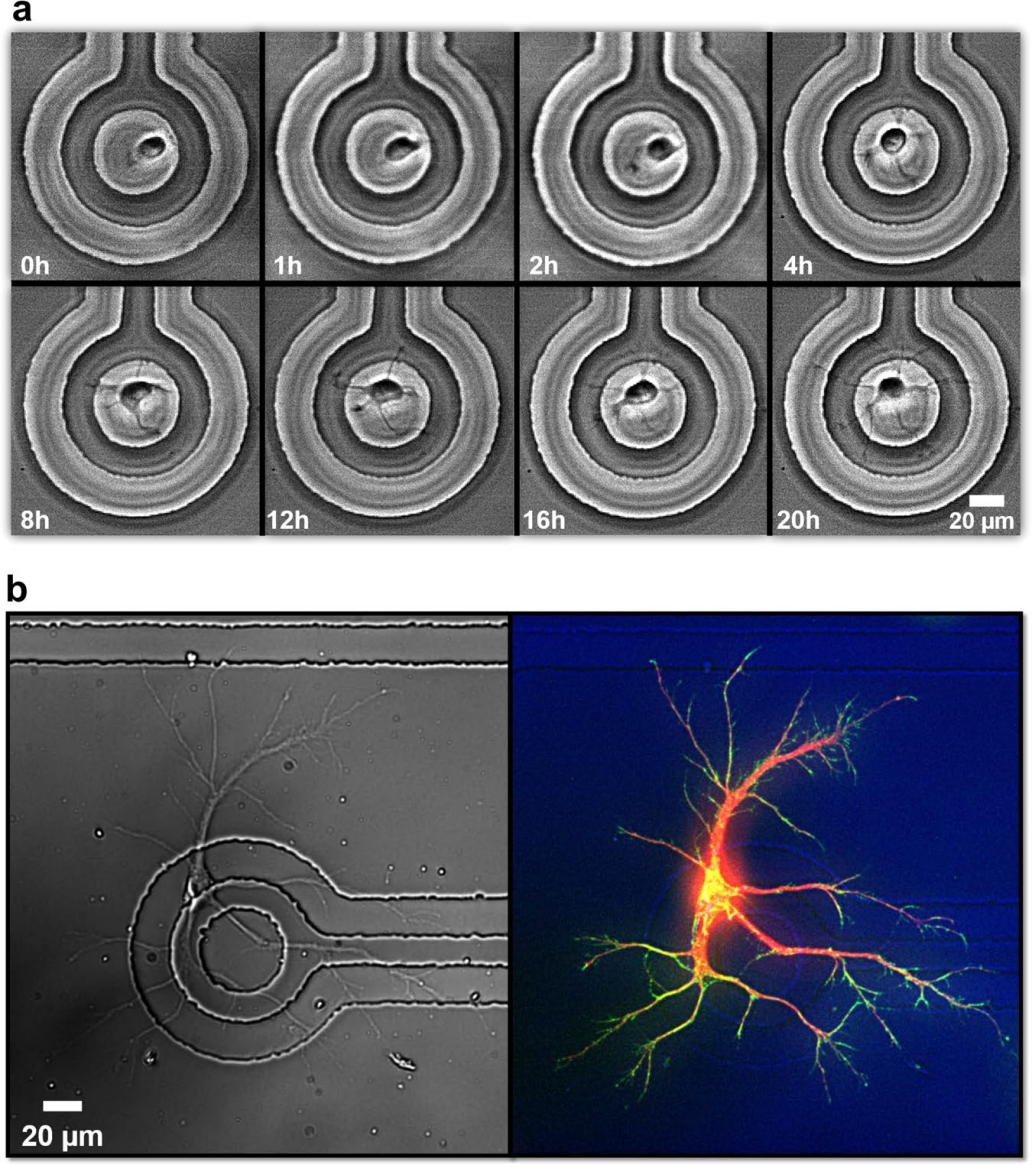
Images of cultured neurons on trap electrodes. (**a**) *In vitro* time-lapse imaging of outgrowth of a single neuron on the trap electrode for 20 h. The trapped neuron was attached on the surface at the initial stage of the imaging. Time-lapse phase contrast images of a living cortical neuron show outgrowth of neurites. (**b**) Microscope image of a cultured neuron on a trap electrode (left). Neuron was fixed at 5 days *in vitro* (5DIV). Image of neuron immunolabeled for microtubules (red) and actin (green) (right).

To confirm whether the trapped single neurons settle and grow well on the trap electrode, we monitored the morphological changes of growing neurons for 20 hrs. Figure 3a shows the time-lapse phase contrast images of neurite outgrowth in an *in vitro* culture of a trapped single neuron. As can be seen in this figure, the morphology of neuron changed slightly after 1 hr and minor neurites began to form after 2 hrs. After 4 hrs, we observed that several minor neurites protruded the cell body and continued to extend over time. These results demonstrate the viability of the technique as we confirmed that a single neuron successfully adhered to the trap electrode and grew well over time.

All of the neurons cultured on the fabricated devices show the same developmental trajectory. First, neurons exhibited a stereotypical series of events in which they first attach to a substrate and extend both lamellipodia and filopodia (stage 1). Over time, filopodia merge and form several distinct neurites, all of which contain a growth cone at their tip (stage 2). Within 48 hours of plating, one of the neurites elongates rapidly to form an axon (stage 3), while the remaining neurites develop slowly into dendrites (stage 4)^27^. A DIC and a fluorescent image of a single neuron cultured on the trap electrode are shown in Fig. 3b. Neurons cultured on poly-_D_-lysine(PDL)-coated DEP devices were fixed at 5 days *in vitro* (5DIV) and labelled for microtubules (red) and actin (green) (Fig. 3b). Phalloidin staining at the tips of both neurites and growth cones show typical stage 2 to 3 development, as well as prominent tyrosinated tubulin staining, which labels dynamic microtubules, in both dendrites and axons^28^.

## Conclusion

We have presented an advanced single-neuronal cell culture and monitoring platform which enables single-neurons to be positioned at a desired location. Further, we conducted real-time live-cell imaging within a controlled environment to monitor the outgrowth of neurons while preventing exposure of the neuronal cells to hostile environments. Finite element simulation was used to guide the appropriate design parameters and verify the two-dimensional model of the proposed structures. Following fabrication of the device, we demonstrated the capability to trap individual neurons on specific target electrodes with our DEP device. Changes in cell morphology, such as neurite outgrowth, were accurately observed through the transparent substrate in phase contrast, while avoiding photodamage to neurons that often accompanies fluorescent imaging. Importantly, the applied electric field for DEP did not adversely affect cell health, as demonstrated by live-cell imaging and immunolabelled images as presented in Fig. 3a,b. Additionally, the accessibility of neurons appropriately grown on the MEAs makes it possible to record the electrical activity of multiple neurons at the same time, and to investigate the electrical communication between them. Therefore, DEP using MEAs allows not only a label-free and non-destructive technique for cell manipulation but also a non-invasive interface with biological cells for long-term (days to weeks) monitoring of electrophysiological parameters^21,29,30^. Thus, the proposed advanced platform has great potential to form an *in vitro* ordered neuronal network and allows novel and detailed studies of cellular physiology.

## Methods

### Fabrication of fully-transparent microfluidic DEP device

**Figure 4:**
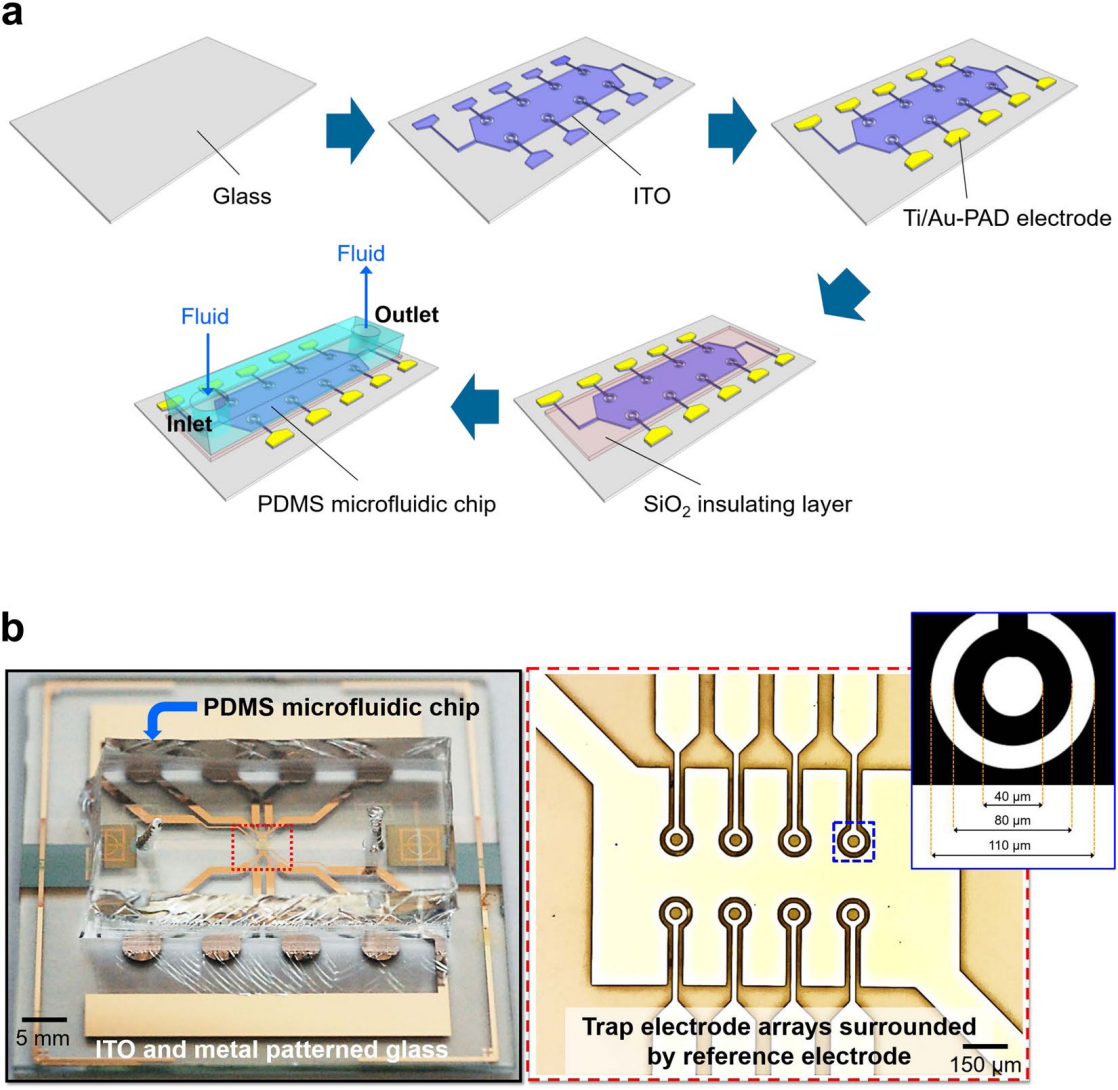
Fully transparent microfluidic DEP device. (**a**) Schematic illustration of the fabrication process of the microfluidic DEP device: ITO patterned to form neuron trapping electrodes. Metal patterning of traces and pads on ITO patterned glass. Electrodes are insulated with SiO_2_ except metal PADs. Alignment and bonding between electrode patterned substrate and the PDMS microfluidic chip. (**b**) Image of the fabricated microfluidic DEP device and optical microscope image of the electrode arrays. Each ring-shaped electrode is surrounded by the reference electrode and connected to the metal pads to apply AC signals.

The proposed fully-transparent microfluidic DEP device is composed of the MEAs integrated glass substrate and microfluidic chip^31^. The fabrication process is described with schematic illustrations in Fig. 4a. The fabrication began with ITO deposition and patterning. A 250 nm thick ITO film with a sheet resistance of 6 Ω/□ was deposited on a clean glass substrate by radio frequency (RF) magnetron sputtering at room temperature. Then, a photolithography and wet-etching processes using hydrochloric acid (HCl) and buffered oxide etchant (BOE) solution were carried out to define the ring-shaped trap electrode and counter electrodes. A bilayer of Ti (20 nm)/Au (200 nm) was deposited using an electron beam evaporator to serve as the pad electrode for making a connection between the DEP device and the signal generator (Fig. 4a). Then, a dielectric insulator (SiO_2_) with a thickness of 200 nm was deposited by an electron-beam evaporator and patterned by the wet-etching process to define the pad electrodes. The internal diameter and the width of the ring-shaped electrode were 40 μm and 20 μm, respectively. The gap between the ring electrode and the ground plane was 20 μm. Other parts of the device, such as the contact pads and traces, were made of metal (Ti/Au). The PDMS-based microfluidic chip was fabricated on a silicon wafer following a previously described soft lithography protocol^32^. The master replica for rapid prototyping of the PDMS microstructure was patterned using negative photoresist (SU-8 50, MicroChem Co., Newton, MA) on a silicon wafer. First, a layer of SU-8 was spin-coated at 4000 rpm. The SU-8 coated wafer was baked and exposed through a photomask containing the desired patterns. After the post-baking treatment, the SU-8 coated wafer was developed leaving master patterns. Liquid PDMS was poured onto the master replica and cured. And then peeled off the cured PDMS from the master replica after 24 hours. The fabricated PDMS chip was oxygen plasma treated and bonded with the target substrate in which MEAs were fabricated to form the microfluidic channel (Fig. 4b). The diameters of inlet and outlet holes in the microfluidic chip were 2 mm. The microfluidic channel height, width, and length were 40 μm, 250 μm, and 3 cm, respectively. Before cells were injected into the microfluidic channel, the fabricated device was cleaned by 75% ethanol and distilled water, and then sterilized by autoclave. After the autoclaving process, all subsequent procedures were performed in a sterile environment. PDL is commonly coated on tissue cultureware to promote surface adhesion to the cell membrane. If the channel was immersed in the PDL solution first, the solution would have hindered at the entrance of the channel due to surface tension^18,21^. Hence, the device was first treated with 95% ethanol for 5 min, followed by rinsing five times with sterile deionized water. Finally, the inside of the microfluidic channel was coated with a PDL (concentration of 0.1 mg/mL).

### System set-up

The DEP device consists of electrode arrays patterned on a glass slide and PDMS microfluidic chip fabricated using standard photolithography and soft lithography processes as shown in Fig. 4a. The device features a total of eight trap electrodes located in the center of the device with the ability to control each electrode independently to trap and release cells. The use of metal (Ti/Au) pads ensures a stable mechanical connection to the cable connectors used for applying the AC signal. Figure 4b shows an image of the fabricated microfluidic DEP device after the PDMS chip was bonded to the MEAs.

**Figure 5:**
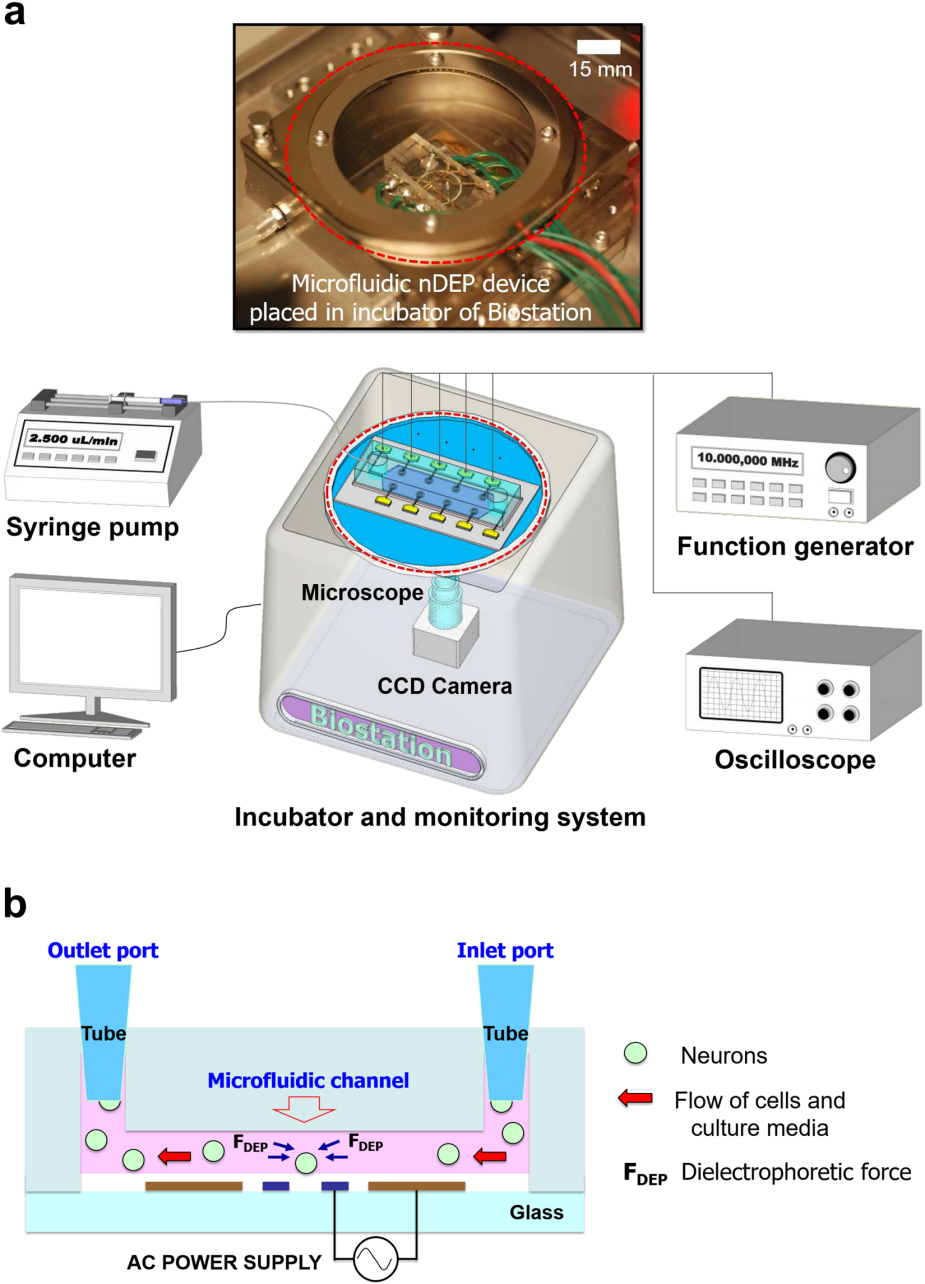
Single-neuronal cell trapping and culture system. (**a**) Overview of the cell incubator and monitoring system, as well as an image of the microfluidic DEP device placed in the incubator. The set-up is composed of a syringe pump, a function generator, an oscilloscope, a camera, a microscope and a monitoring computer. Each metal pad of the eight ring-shaped electrodes is connected to the positive terminal of the function generator and the two reference electrodes are connected to the ground terminal of the function generator. (**b**) Schematic illustration of the cross-section of the microfluidic DEP device.

A schematic of the overall experimental set-up for single-neuronal cell trap and culture is depicted in Fig. 5a. The fabricated microfluidic DEP device was placed in the incubator that incorporates a motorized inverted microscope (BioStation, Nikon, Inc.). and a CCD digital camera (DS-Qi1, Nikon, Inc.) to facilitate live-cell imaging. A mixture of cell culture media and neurons was loaded onto a 1 mL syringe and the needle inserted into the inlet tube (6.25 × 10^−2^ inch inner diameter) connected to the microfluidic channel designed to flow the mixture. The syringe was placed in a syringe pump (Kent Scientific, Genie plus, CT) set a flow rate of 2.5 µL/min. The AC signal used to trap cells on the electrodes was generated by a function generator (HP 33120 A) and its amplitude and frequency were 8 V_pp_ and 10 MHz, respectively. In order to prevent signal attenuation, AC signal was applied to the electrode via RF coaxial cable connectors (Taoglas Limited CAB.058 semi-rigid SMA RF connector), by which impedance was matched to 50 ohms, and was confirmed by the signal measurement using an oscilloscope (Agilent 54621 A). Operation of the microfluidic DEP device is depicted in Fig. 5b. When the target neuron approaches the trap, the electrode is energized to immobilize the neuron at the center of the electrode. After neurons are positioned inside the electrodes, a medium without neurons was introduced into the microfluidic channel to remove the excess cells. Neurons attached to the target electrodes after the medium stopped flowing. The immobilized neurons were cultured and the growth of the neurons was recorded in the incubator.

### Cortical neuron culture

All animal procedures were approved by the University of Wisconsin Institutional Animal Care and Use Committee (IACUC) and were in accordance with National Institutes of Health guidelines. Embryonic day (E) 18 cortical/hippocampal neuron cultures were prepared from Sprague-Dawley rats of either sex (Envigo) as described previously^33^. Briefly, cortices were dissected, trypsinized and dissociated. Dissociated cortical neurons were plated on 1.0 mg/mL PDL-coated DEP device. Neurons were plated in plating media (PM) (Neurobasal medium with 5% FBS (Hyclone), B27 supplement, 2 mM glutamine, 37.5 mM NaCl and 0.3% glucose). After 1 h, the medium was replaced with serum-free medium (SFM), which was PM without FBS. Neurons were then fixed and imaged after 5DIV.

### Immunocytochemistry and imaging

For wide-field imaging, cortical neurons were fixed in 4% paraformaldehyde/Krebs/Sucrose at 37 °C. Cultures were rinsed three times with phosphate buffered saline (PBS) solution and blocked with 10% BSA/PBS, permeabilized in 0.2% Triton X-100/PBS and labelled with primary and secondary antibodies. Primary antibodies to the α-tubulin, specifically, Tyrosinated-Tubulin (Millipore) and Tau-1 (Chemicon) and secondary antibodies to goat anti-rat and goat anti-mouse IgG Alexa Fluor 488, 568 and 647 (Invitrogen) were used to visualize microtubules. Phalloidin coupled to Alexa 488, 568 or 647 (Invitrogen) was used to label actin filaments (1:25 to 1:100). Neurons were imaged on a Nikon TE300 inverted microscope equipped with a 40X/1.3NA Plan Apo (DIC-fluor) and 20X/0.5NA (phase-fluor) objective. Images were captured on a Coolsnap EZ cooled interline CCD camera (Photometrics).

## Electronic supplementary material

Movie S1: Single-neuronal cell manipulation

## Data Availability

The data supporting the findings of this study are included within the paper and its Supplementary Information, or available from the corresponding authors upon reasonable request.
